# Managing MMP-2, MMP-9, VEGFR-2, TGFβ-1, and TIMP-1 in NNK-induced lung carcinoma by nonchemical interventions in female rats

**DOI:** 10.1016/j.toxrep.2022.05.018

**Published:** 2022-05-30

**Authors:** Zahra Abrishami Kia, Seyede Tayebeh Sadati Bizaki, Elham Asaádi Ghareh Tapeh, Shadmehr Mirdar Harijani, Roya Gorji Baziary

**Affiliations:** a Department of Exercise Physiology, Faculty of Physical Education and Sport Sciences, University of Mazandaran, Babolsar, Iran; b Athletic Performance and Health Research Center, University of Mazandaran, Babolsar, Iran

**Keywords:** MMP-2, MMP-9, *Nigella sativa*, NNK, Submaximal swimming exercise, TGFβ-1, TIMP-1, VEGFR-2

## Abstract

**Background:**

Smart and flexible methods are attracting remarkable interest in cancer-related biological and chemical therapies. To achieve a safer, affordable, and more effective cancer treatment, we evaluated the application of submaximal swimming and *Nigella sativa* (NS) nano-drug on lung tissues of female rats induced by NNK.

**Material and methods:**

A 12-weeks protocol of submaximal swimming was performed in pathologic and non-pathologic groups. NNK and NS groups, respectively received weekly doses of 12/5 mg/kg and 125 μg/kg of body weight. By the end of the protocol, the ratios of MMP-2, MMP-9, and TIMP-1 determined by using immunohistochemistry essay, and RT-PCR analysis for VEGFR-2 and TGFβ-1.

**Results:**

As a result, treatment with exercise and NNK resulted in VEGFR-2 overexpression (*P ≤ 0.001* and *P ≤ 0.05,* respectively). In NNK, NNK+E, NNK+NS, and NNK+NS+E groups, protein expression of MMP-2 and MMP-9 significantly increased, despite the reduction of TIMP-1 levels in the same groups compared to control *(P ≤ 0.001).* TGFβ-1 ratio significantly increased following preformed interventions in non-pathologic groups: E (*P ≤ 0.001)* and NS+E (*P ≤ 0.01)*.

**Conclusion:**

IHC and gene assays indicate a favorable and acceptable effect of the designed training protocol besides the treatment with *N.sativa* nano-drug, by which cancer development could be restricted through recovering the natural balance of angiogenic and angiostatic markers.

## Highlights

• NNK was used to induce the primary lung cancer in experiments.
• Submaximal swimming and *N. sativa* nanocapsule were applied in rats induced by NNK.
• The effects of operating parameters were investigated.
• At the optimum operational condition, submaximal swimming and *N.sativa* exhibited excellent performance on measured markers.

## 1 Introduction

Lung cancer incidence with about 15ˌ000 deaths annually ranks first as the most prevalent and fatal cancer worldwide [1]. According to epidemiologic evidences, 90% of lung cancer-related deaths occur through active or passive exposure to the tobacco smoke. Among Tobacco carcinogens such as polycyclicaromatic hydrocarbons, nitrosamines, and aromatic amines, 4-(methylnitrosamino)− 1-(3-pyridyl)− 1-butanone (NNK) has demonstrated to have a remarkable association with the lung cancer incidence, development, and progression through directly disrupting many physiological signaling pathways and DNA damages, adduction and mutation [2]. [2] Based on oncogenic studies, several cytochrome P450s activate NNK, through some distinct reactions such as α-hydroxylation, pyridine oxidation, and carbonyl reduction will produce α-hydroxylation (α-hydroxy NNKs and α-hydroxy 4-(methylnitrosamino)− 1-(3-pyridyl)− 1-butanol (NNAL)) and DNA adducts [3]. The lack of rapid DNA repair leads to permanent gene mutations, uncontrolled cell proliferation, and transformation. Additionally, other NNK-mediated cell proliferation, survival, and angiogenesis mechanisms such as phosphatidylinositol-3-kinase (PI3K)/protein kinase B (PKB/AKT), protein kinase C (PKC), and nuclear factor kappa B (NF-κB) will contribute to the development of smoking-associated lung cancers [4].

Under cancer condition, dysregulated expressions of various cytokines, angiogenic and angiostatic markers form tumor progression. Multi-functional cytokine VEGF and its major receptor VEGFR-2 have key role in imbalanced angiogenesis within the tumor tissue [5]. The genetic deregulation of VEGFs cause disorganized blood vessels, and results in impaired or overgrowth endothelial cells [6]. Development of tumor invasion and metastasis is firmly associated with the actions of two proteases: MMP-2 and MMP-9 which are identified in various types of human cancers and also inflammatory cells. A wide distribution of matrix metalloproteinases MMP-2 and MMP-9 (gelatinase B) are respectively expressed by endothelial cells, inflammatory cells and connective tissues [7]. Previous studies have demonstrated a positive correlation between cancer progression and the presence of MMPs [8], [9]. On the other hand, the actions of some proteins, such as low weight endogenous tissue inhibitors, TIMPs, suppress MMPs expression through forming complexes of activate enzymes. The production of MMPs and TIMP-1 in lung cancer tissue derived cell lines, represented cancer clinical behavior. It seems that the balance of TIMPs and MMPs leads matrix turnover and ECM degradation [10]. In metazoan biology, the role of regulatory cytokine TGFβ-1 signaling pathway is substantial, however its dysregulation results in tumor development. This marker mediates cell invasion, immune regulation, and microenvironment modification that cancer cells may exploit to their advantage. Paradoxically, this marker exerts tumor-suppressive effects that cancer cells must activate for malignant evolution [11]. In many cell types, for instance, TGFβ-1 induces angiogenesis through strongly upregulating the expression of VEGFs and also inhibiting the proteolytic activities, endothelial cells proliferation, and migration [12], [13]. Even though, the dual role of TGFβ in cancer studies has long been noted, but its mechanistic basis and clinical relevance have remained elusive.

The relation between cancer and exercise therapy continues to evolve. It is confirmed that regular exercise training is associated with the reduced hallmark or mortality rate of developing lung cancer as well as colon, breast, esophageal, pancreas, endometrial, and ovarian cancers [14].

Based on population-based studies, exercise intervention is capable to protect the host through boosting immunity and reducing chronic inflammation, both of which are reported to reduce carcinogenesis process [15]. Plausible mechanisms of exercise training include the activation of DNA repair enzymes and exercise-induced antioxidants that decrease oxidative stress level [16]. That’s why tumor-mitigating properties of exercise training across a spectrum of malignancies are taken into researcher’s consideration. However, the exact molecular mechanisms behind exercise clinical findings are not fully understood.

in the other area of cancer treatment studies, various biological active components of planet *N.sativa* are utilized in pharmacology [17]. This miraculous planet with the power of healing, antidiabetic, antimicrobial, anti- inflammatory, and anti-malignancy has got the place among the top ranked evidence based herbal medicine. Nanotechnology gives the chance to perfectly deliver drugs with reduced toxicity, improved drug targeting, and reduced drug dose frequency. In cancer studies, nanoparticles synthesized form of *N.sativa* had shown great effectiveness and acceptable treatment results [18]. Since *N.sativa* is a natural biocompatible compound, its proper consumption besides posing no danger to normal live cells, amino groups, oils, and essential oils make it capable in restricting malignant tissue progression.

To our knowledge, this IHC and RT-PCR analysis on NNK-induced lung tissues of female rats, provides the first evidence in support of the hypothesis that a 12-weeks protocol of submaximal swimming and 125 μ/kg of *N.sativa* nanocapsule manage protein and gene expression of VEGFR-2, MMP-2 and MMP-9, TIMP-1, and TGFβ-1 through regulatory mechanisms in lung tissue induced by NNK.

## 2 Material and methods

### 2.1 Experimental animals

72 Female Wistar rats aged 6–8 weeks and weighted 105.84 ± 27.93gr (Samurai Techno weight India Pvt. Ltd) were obtained from Pasteur Institute of Iran. The animals were kept in polycarbonate cages, 12/12 h light/dark cycle with free and sufficient access to standard pellet diet and water ad libitum. The housing and treatments of animals were in accordance with the Guide for the Care and Use of Laboratory Animals published by the US National Institutes of Health (NIH Publication, 8th Edition, 2011) and approved by HRI Ethics Committee of the Babol University of Medical Sciences (Code No: MUBABOL.HRI.REC.1395.109).

4-(Methylnitrosamino)− 1-(3-pyridyl)− 1-butanone (NNK) with 207/23 molecular weight was obtained from TRC, North York, Ontario, Canada. This carcinogen is available in form of a pale yellow crystalline solid which is easily soluble in any organic solvent. Commercial seeds of *N.sativa* were obtained from a local market located in Babolsar, Iran. The variety of seeds was confirmed by the herbarium official expert of University of Mazandaran. The voucher specimens containing seeds were transferred in the laboratory of exercise physiology department for the following process. Animals were divided in 9 groups of 8 animal each and assigned as: Control (C), Solvent (S), (NNK), *N.sativa* (NS), Exercise training (E), *N.sativa*+ Exercise (NS+E), NNK+Exercise training (NNK+E), NNK+*N.sativa* (NNK+NS), NNK+Exercise+*N.sativa* (NNK+NS+E).

NNK was IP injected for 12 weeks as 12/5 mg/kg [19]. Additionally, rats of *N.sativa* nano-capsule groups received weekly IP injection doses of 125 μg/kg [20]. The solvent group received the same amount of normal saline (0.9% NaCl) on the same schedule [19].

### 2.2 Preparation of herbal extract

The primary *N.sativa* extract was prepared by using the soaking method. This method is suggested to preserve and extract the major active or inactive components of *N.sativa*. The seeds were manually battered with mortar and pestle. 10gr of the powder was mixed with 20 ml of PBS (phosphate-buffered saline), then whipped consistently until attaining solution with a preserved color. The obtained solution was kept at 4 °C in sterile tubes overnight and centrifuged at 3000 rpm for 15 min. To purify the brownish orange color supernatant a Whatman paper filter was used. The solution was restored in disinfected micro tubes at 4 °C until consumption. Finally, the total proteins were quantified by using the Bradford reagent, and a DU-70 spectrophotometer used for spectrophotometry [20].

### 2.3 Preparation of *N.sativa* nanocapsules

To produce *N.sativa* nanocapsule, 50 mg of human albumin serum was dissolved in 1 ml of water pH: 7.4. Tween 80 (0.5% v/v) was delivered to the samples, then stirred 30 min at 500 rpm using a magnet. 4 ml Ethanol was added into the stirring solution. 117 ml of Glutaraldehyde was surcharged to the samples to produce particle cross-likings, then stirred continuously at 500 rpm for 24 h. After that by adding *N.sativa* extract to the solution, its shape remained spherical and formed slightly, as the indicator of the synthesized nanocapsules containing *N.sativa* extract [20]. The extracted *N.sativa* nanocapsules were injected subcutaneously at the weekly dose of 125 milligrams per kilogram of body weight during a 12-week protocol.

### 2.4 Training protocol

Two weeks before the main protocol, the rats got familiar with exercising during short sessions of swimming programs. The designed swimming protocol was 5 days/week and 20 min/session. Rats swam in a 50 × 50 × 100 cm dimensioned pool, and temperature of 30–32° [21]. Then, blood lactate threshold was measured in 4, 7, 10, and 13 liter/min intensities (Lactate Scout 4; EKF Diagnostics Company, Germany). Regarding the lactate levels, the power of water arranged in submaximal levels 4–10 L/min intensities [22].

### 2.5 Tissue sample preparation

48hrs after the last training session, the rats were weighted and anesthetized by ketamine (50 mg/kg) and xylazine (40 mg/kg) [23]. The lung tissues were removed by thoracic surgery, rinsed with physiological serum, isolated and then kept in the micro tubes in a LN2 freezer at - 80 °C in liquid nitrogen for following gene expression analysis. Left lobes of lung tissues were fixed by 5% formaldehyde for subsequent IHC analysis.

### 2.6 Immunofluorescence method for IHC detection of MMP-2 and MMP-9, and TIMP-1 in lung tissue of male Wistar rats

All steps of IHC analysis were assigned based on the manufacturer's instruction. After dewaxing paraffin-embedded blocks, endogenous peroxidases were deactivated through 3% H2O2. Samples were pre-incubated with 10% goat serum, after thermal remediation with citrate buffer, Then, diluted primary antibodies (1: 100) (MMP-2 antibody: AB37150; MMP-9 antibody: AB38898, and TIMP-1 antibody: AB61224) were added to the samples. The samples were incubated at 4 °C overnight and then washed for three times with PBS buffer. A peroxidase-labeled polymer (secondary antibody; ab97050) was used to coat the samples for 30 min and then washed with PBS for three times. In the end, DAPI staining solution (1:100) (ab228549) was added to samples and then washed with PBS. Finally, pictures were taken under Olympus fluorescent microscope with 400 lenses to confirm MMP-2, MMP-9, and TIMP-1 expressions (Fig. 2 A-C) [24].

**Fig. 1:**
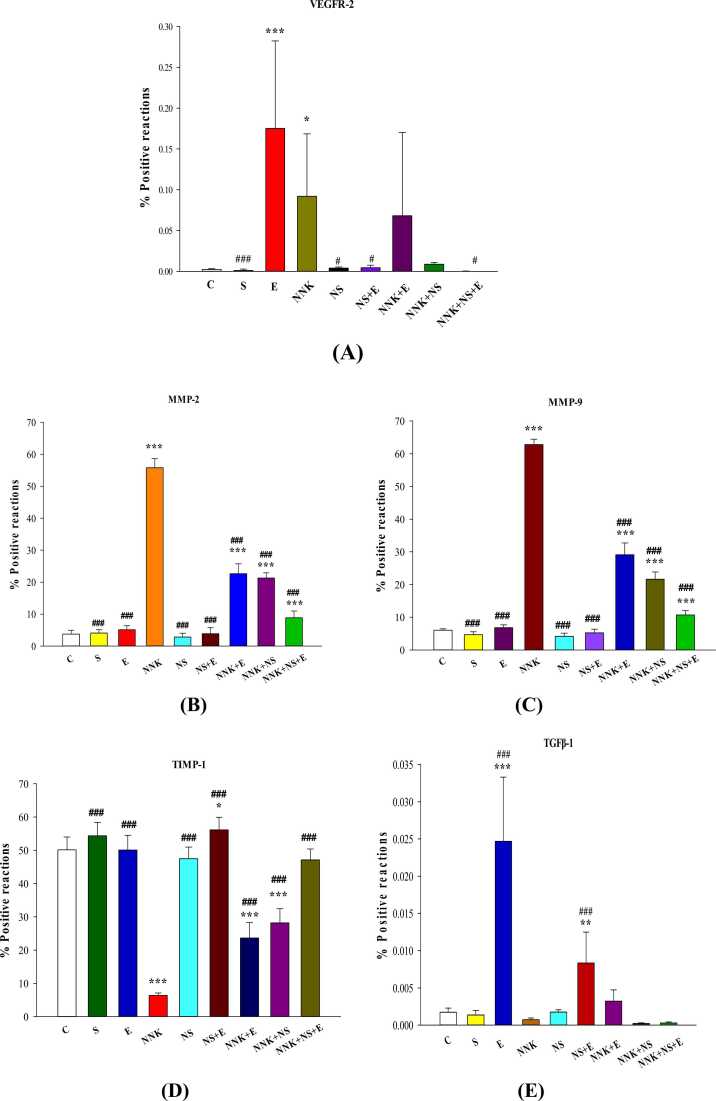
The results of statistical analysis (mean ± SD). **A**: VEGFR-2, **B**: MMP-2, **C**: MMP-9, **D**: TIMP-1, and **E**: TGFβ-1 levels in lung tissues of experimental groups (n = 8). The Statistical significances for the difference between the data of experimental groups and control group are demonstrated by * (*P ≤ 0.05),* **(*P ≤ 0.01)*, and ***(P ≤ 0.001). The Statistical significances for the differences between the data of experimental groups and NNK group are demonstrated by # (*P ≤ 0.05),* ## (*P ≤ 0.01)*, and ### *(P ≤ 0.001).*

**Fig. 2:**
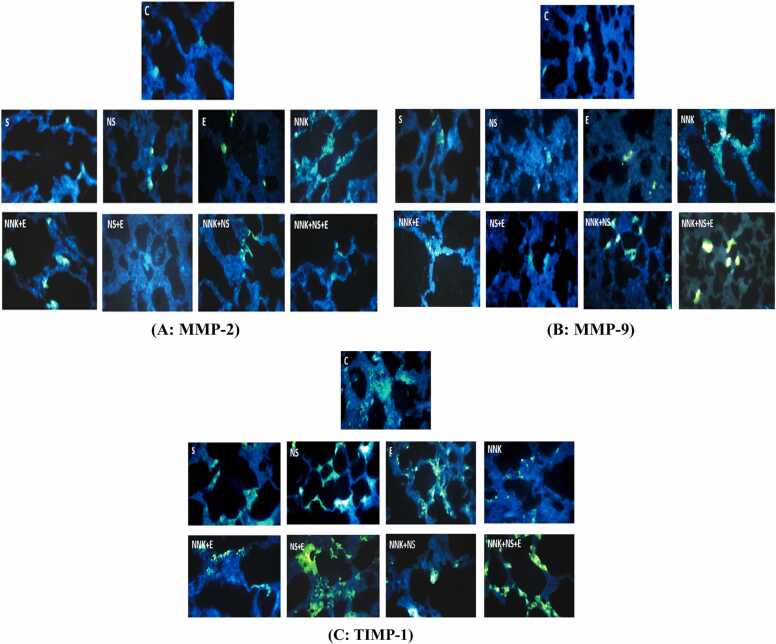
Immunohistochemistry (IHC) detection of MMP-2, MMP-9, and TIMP-1 in lung tissues of studied groups by immunofluorescence. The sections were incubated with 10% goat serum and diluted with **A:** MMP-2 antibody: AB37150, **B:** MMP-9 antibody: AB38898, and **C:** TIMP-1 antibody (AB61224). n = 8 each group. Blue color refers to nuclei stained by DAPI. Green color refers to primary antibodies of studied targets. Differential staining of hematoxylin with magnification 400 x.

### 2.7 Gene expression (TGFβ-1, and VEGFR-2)

Quantitative RT-PCR method was performed to evaluating VEGFR-2 and TGFβ-1 genes expression in lung tissues of female rats. In order to isolate the lung RNA, TRIzol Reagent (Thermo Fisher Scientific, Inc., Waltham, MA, USA) and first-standard cDNA synthesis (Thermo Fisher Scientific, Inc., Waltham, MA, USA) were used based on manufacturer's guidelines.

Expression ratios of VEGFR-2 and TGFβ-1 were detected by RT-PCR assay using GAPDH as an internal control. The following primers used for real-time PCR for VEGFR-2 were: forward 5′-ACAGAGAACAAGGACACACTCAC-3′, reverse 5′-ATGTAGCACGACAGAGACTGTGA-3′, for GAPDH control, forward 5′-AAGTTCAACGGCACAGTCAAGG-3′, and reverse 5′-CATACTCAGCACCAGCATCACC-3′. The primers used for real-time PCR assay for TGFβ-1 were: forward 5′- GCAGTGGCTGAACCAAGGAGACG3', reverse 5′ GTCGGTTCATGTCATGGATGGTG3', for GAPDH control, forward 5′-AAGTTCAACGGCACAGTCAAGG3′ and reverse 5′CATACTCAGCACCAGCATCACC3'. Quantitative values of VEGFR-2 and TGFβ-1 were obtained from the threshold cycle value (Ct), and the relative expressions of VEGFR-2 and TGFβ-1 genes which were calculated using the ΔΔCT method [25].

### 2.8 Statistical analysis

Data analysis were followed by Tukey post hoc test and was performed as mean ± SD and medians 95% confidence, in SPSS version 26.0 (SPSS Inc., Chicago, IL, USA), and graphed in sigma plot version 14.0. The significance level was adjusted to 5% *(P ≤ 0.05).* One-way ANOVA was assessed, to determine the results of differences groups with control and solvents groups.

## 3 Results

Rats were weighted at the end of the protocol as well as the arrival day. There was any significant difference among the body weights of different experimental groups *(P > 0.05)*, while all groups showed a significant increase in body weight amounts compared to theirs of the arrival weight measurement *(P ≤ 0.05).* According to Fig. 1A, there was a significant increase in the levels of VEGFR-2 in E *(P ≤ 0.001)* and NNK *(P ≤ 0.05)* groups versus control group. Additionally, VEGFR-2 levels of NS, NS+E, NNK+NS+E groups significantly decreased compared to NNK group (*P ≤ 0.05*). MMP-2 and MMP-9 protein levels were significantly expressed higher in NNK, NNK+E, NNK+NS, NNK+NS+E groups compared to control groups (*P ≤ 0.001*). These markers remarkably decreased following preforming E, NS, NS+E, NNK+E, NNK+NS, and NNK+NS+E compared to NNK group *(P ≤ 0.001)* (Fig. 1B and C).

Despite the expression levels of angiogenesis markers, NNK induction significantly reduced the levels of TIMP-1 levels of NNK, NNK+E, NNK+NS (*P ≤ 0.001*), as well as NS+E (*P ≤ 0.05*) versus control. In comparison with NNK group, TIMP-1 levels significantly increased in all the experimental groups (*P ≤ 0.001*) (Fig. 1D). According to the results shown in Fig. 1E, there was a dramatic increase in the levels of TGFβ-1 in E and NS+E group compared to control and NNK groups (*P ≤ 0.001*).

### 3.1 Descriptive analysis of histological results (MMP-2, MMP-9, and TIMP-1)

In this study, we used immunofluorescence double-staining to determine MMP-2, MMP-9, and TIMP-1 expression in the lung tissues of female rats (Depi staining, 400x magnification) (Fig. 2A-C). IHC assessed the expression of each marker quantitatively. Normal structure of lung tissue is visible in IHC photos of all control and solvent groups. Based on histological evaluation, in E groups, parenchymal structure was mostly preserved and there was not any acute inflammation or hyperplasia within the lungs. In rats treated with *N.sativa* and exercise (NS+E), besides the perfectly remained normal parenchymal structure, increased interstitium volume was observed in normal areas of lung tissues. Also mild hyperplasia especially around arteries and bronchioles, small quantity of inflammatory cells, slight deformity in parenchymal tissue, and very small areas of alveolar changes were visible in NS+E groups. The connective tissues around the airways and arteries in NNK groups had obviously increased amounts of neutrophil and plasma cells. In addition, increased interstitium volume, emphysema and the reduced volume of alveolar space were observed. The major features of lung tissues exposed to NNK were accumulation of atypical cells, inflammatory cells, hyperplasia (the arrows are the signs of atypical cells), and small foci of a seemingly tumorous areas. NNK+E groups had relatively preserved parenchymal structures, despite the acute inflammation and mild hyperplasia. Foci of acute inflammation in NNK+E implied the reduced alveolar volume and emphysematous alterations. In the NNK+NS groups, mild inflammation and altered parenchymal tissues were observed, however the alveoli structures were well-preserved. According to Fig. 2A-C, there was a mild inflammation in NNK+NS+E groups, and the parenchymal structure of the lung tissue was often preserved.

## 4 Discussion

The potent carcinogen NNK has been found to effectively induce various types of lung cancer. It has been reported that different methodologies of dosages and routs of NNK induction in mice, rat, hamster, and ferret have resulted in lung cancer. 100 mg/kg intraperitoneal (i.p.) injection of NNK, after 14 weeks led to the hyperplasia along the lung alveolar septa. Following that, in 54th week of the same protocol, pulmonary lesions developed carcinoma was observed [26]. Developed lung adenoma was also observed following twenty-six-week i.g. (intragastric gavage) and i.p. induction on NNK in female A/J mice [27]. NKK induction in mouse, after 24 weeks, resulted in 12 of 66 progeny and 13 of the 14 mothers with developed lung tumors [28]. Numerous lines of evidence have previously established the mechanisms of actions of metabolized NNK to induce several kinds of cancer in different animal models. Carcinogen-mediated DNA mutations and abnormal signaling pathways which are activated by associated receptors, such as α7 nicotinic acetylcholine receptor (α7-nAChR) and β-adrenergic receptor (β-AR) are the major underlying mechanisms of NNK leading to malignancies. Since nAChRs are low O2/ high CO2 environment sensitive, NNK prefers to bind to the nicotinic acetylcholine receptors in lungs of smokers, in which the expiration of CO2 has decreased [1]. This connection, finally stimulates neural cell adhesion molecule (Contactin 1) which interacts with several other membrane proteins or extracellular matrix to activate the downstream signaling pathways following NNK exposure. Contactin 1 promotes tumor invasion and metastasis induced by VEGF-C/Flt-4/Src/p38 MAPK/C/EBPα pathway. NNK-stimulated angiogenesis and cell survival pathways will arise following the activated arachidonic acid metabolism pathway, more synthesized TxA_2_, and activated downstream PI3K/AKT/CREB pathway. NF-κB and ERK1/2, the other NNK-stimulated pathways, will induce HO-1 expression that correlates with tumor invasiveness, advanced stages, and poor prognosis[29].

It is known that NNK is a relatively specific lung carcinogen. Interestingly, our results demonstrated that, the dose of 12/5 mg/kg NNK injection in female rats resulted not only in increased angiogenesis process of lung tissue, but also its injection significantly decreased the amount of TIMP-1, besides the approximate steady levels of TGFβ-1 versus control group (Fig. 1A-E). In this regard, many studies which were looking for reliable prognostic factors have been tested these enzymes. Some studies reported the association between increased levels of MMP-2, MMP-9 and decreased TIMP-1 in early stage lung cancer [30]. The synthesized MMPs from VEGF-stimulated endothelial cells, destroy the extracellular membrane matrix and activate the endothelial Tip cells leading tumor angiogenesis [31]. The increased MMPs in NNK-induced lung tissue, evaluate invasion and affect other downstream angiogenic and anti angiostatic factors [9]. Similarly, it seems that, under pathologic conditions of this study, the endothelial cell vesicles containing MMPs, through stimulating VEGF upregulated the angiogenesis pathways. The increased gene expression of VEGFR-2 in NNK group, subsequently, provided the optimal culture for more ligand (VEGF) binding resulted in MMPs pathologic overexpression [32], [33]. Cancer neovascularization is initiated by alteration in the balance between pro- and anti-angiogenic molecules in the tumor microenvironment [34].

Inefficient actions of suppressors TIMP-1 and TGFβ-1 besides uncontrolled angiogenesis, might have intensified the pathogenicity of 12/5 mg/kg of NNK induction in female rats. In long-term NNK exposure, pathologically activated TGF-β, TGFβ-TGFβR-SMAD2 signaling altered alveolar epithelial cell features, mitochondrial dysfunction and apoptosis. Moreover, TGF-β through SMAD, DAXX/HIPK and TAK1/TRAF6 signaling pathways, induced apoptosis and caused cell death, and tumor progression [13]. Although increased TGFβ-1 synthesis was observed in previous reports of different types of advanced lung cancer models, but on the contrary, in some primary lung cancer studies reduced or steady levels of this cancer promoter/suppressor marker was reported along with increased cell matrix interactions, suppressed immune surveillance, or increased angiogenic activity. Since TGFβ-1 gene, mRNA, and protein are mostly secreted by macrophages and fibroblasts of tumor stroma, steady levels of this dual-action marker, might be an index of the activity of the primary cancer signaling following a short duration of NNK induction in female rats, before the formation of tumor masses, as observed in the present study [35], [36] (Fig. 1E).

An important phenomenon described in the literature of anti-cancer studies is blocking the major angiogenesis regulator: inflammation, via utilizing various interventions [37]. In this study, we applied IHC and gene assays on some of angiogenic and angiostatic markers to investigate the potential effects of *N.sativa* nanocapsule, submaximal exercise training, and a combination of them on NNK-induced lung tissue.

As demonstrated in Fig. 1A, in addition to the predictable pathologic over expression of VEGFR-2 following NNK exposure, there was a meaningful increase in natural physiologic gene expression of VEGR-2 in E group. Increased levels of VEGF-A, VEGFR-1 and VEGFR-2 mRNA expression were reported following submaximal training bouts. Low tissue oxygen tension (hypoxia) and increased muscle capillary density are well-established and functionally important adaptation of submaximal exercise which directly trigger angiogenesis [38]. Moreover, In NNK-exposed rats, despite the inefficacy of single-preformed intervention (NNK+E and NNK+NS groups) to recover VEGFR-2 levels compared to NNK group, combined intervention (NNK+NS+E) could remarkably decrease VEGFR-2 gene expression compared to NNK group.

Referred to Fig. 1B and C, MMP-2 and MMP-9 levels of NNK, NNNK+E, NNKK+NS, and NNK+NS+E groups significantly increased compared to control subjects. Even though our interventions were not able to restore base values of MMPs, but the pathologic angiogenesis in the named groups was significantly reduced compared to NNK group. In cancer induced rat models, treatment with *N.sativa*, dysregulated MMPs via reduction of wnt/b, NF-κB, IL-1, and suppressed MAPK signaling pathway [39]. It is interesting to note that, in all the present pathologic and non-pathologic treatment groups, the level of suppressor TIMP-1 was significantly higher than NNK group (Fig. 1D). It seems that, performed interventions could effectively recover the balance of MMPs-TIMP complex. In this context, it has been reported that thymoquinone consumption moderated the levels of IL-6 and subsequently suppressed MMP-1, MMP-3, VEGF, and decreased TIMP-1 in Leukemia. Moreover, in cancer patients exercise training recovered angiogenesis/ angiostatic balance, and increased the action of angiostatic markers. Acute exercise training also increased TIMPs mRNA, and reduced the levels of MMPs via altering eNOS and producing NO [40]. In addition, in NNK-induced rats, exercise adaptation reduced oxidative stress through down regulated NF-κB and over expressed TNF-α. It seems that, swimming exercise through increasing apoptosis markers, contrast the possibility of tumor-genesis induced by NNK [41]. We observed that, injection of *N.sativa* nanocapsule, intensified the anti-tumorigenesis ability of exercise training in this study. This natural component by suppressing the AKT/ERK and reducing NF-κB through STAT signaling pathway, blocked NNK-induced tumor-genesis [42].

During early tumor outgrowth, TGFβ-1 acts as a tumor suppressor, but becomes a tumor promoter at later stages through enhancing tumor cell mobility, invasion and metastasis. Thus, any intervention in early stage cancer must be performed cautiously. The phosphorylation of mutant p53 at Ser6/Ser9 by Ras/MAPK signaling is a crucial hallmark for TGF-β to switch to become a tumor promoter. *N.sativa* [43] and exercise training [44] are known as stimulators of p53 which can indirectly affect the expression behavior of TGFβ-1. In the present study, treatment with *N.sativa* and exercise training (E and NS+E groups), remarkably increased the gene expression of TGFβ-1 in healthy rats (E and NS+E) versus control subjects (Fig. 1E). It is still controversial when and how the exercise interventions and inhibitors should be applied to perfectly suppress TGFβ-1 tumor-promoting signaling. TGFβ-1 characteristics in early stage lung cancer of this study was represented with unchanged steady levels of this marker in NNK group compared to control. In other pathologic groups the amounts of this dual action marker was not significantly affected by exercise and *N.sativa* interventions. Besides the stage of the disease, the lack of leaving significant effects following treatment with applied interventions might be accorded to the intensity, the duration, or the type of exercise training, and also the injection dose of *N.sativa* nanocapsule in NNK-induced female rats. Probably, other exercise patterns and various dosage of *N.sativa* nanocapsule can differently affect this marker. To clarify the fluctuations and the role of TGFβ-1 signaling as a cancer promoter or suppressor, the gene analysis of TGFβ-1 in tumor cells seems to be inadequate. Based on our results, other assessment methods such as western blot are needed to be performed on this controversial marker.

## 5 Conclusion

Summing up, we demonstrated the NNK-pathogen reduction or inactivation in lung tissues of female rats as the consequence of a 12-week protocol of submaximal swimming program and 125 μg/kg i.p. injection of *N.sativa* nanocapsule. According to current immunohistological assessments, these intervention could manage and normalize the NNK-induced lung tissue microenvironment and reduce the lung cancer risk. Such findings may have valuable implications to inhibit tumor metastasis and improving cancer therapies. More mechanistically based researches are now required to gain further understanding of the exact effects and underlying molecular mechanisms of submaximal swimming and *N.sativa* nanocapsule on damaged malignant lung tissue, along with other anti-cancer treatments.

## Funding

This work had no financial support.

## Statements of ethics

All the animal study protocol was approved by the HRI institutional Ethics Committee of 10.13039/501100005716Babol University of Medical Sciences and conformed to I.R.I national institutes of health guidelines (ethics code: MUBABOL.HRI.REC.1395.109).

## CRediT authorship contribution statement

**Zahra Abrishami-Kia** was involved in the designing, execution of experiments, data analysis and proofreading of the manuscript. **Seyede Tayebeh Sadati-Bizaki** was involved in the designing of the study and execution of experiments. **Dr. Shadmehr Mirdar Harijani** was involved in monitoring the methodology. **Elham Asaʹadi Ghare-Tapeh** was involved in data analysis. **Roya Gorgi Baziary** was involved in the designing of the study.

## Declaration of Competing Interest

The authors declare that they have no known competing financial interests or personal relationships that could have appeared to influence the work reported in this paper.
